# Focal Muscular Vibration During Intensive Rehabilitation in the Treatment of Spasticity After a Severe Acquired Brain Injury: A Pilot Randomized Controlled Trial

**DOI:** 10.3390/jcm15093363

**Published:** 2026-04-28

**Authors:** Augusto Fusco, Dario Mattia Gatto, Davide Giannuzzi, Letizia Castelli, Gianpaolo Ronconi, Claudia Lombardo, Stefano Bonomi, Cristina Cuccagna

**Affiliations:** 1 High-Intensity Neurorehabilitation, Department of Neuroscience, Sensory Organs and Thorax, Fondazione Policlinico Universitario “A. Gemelli” IRCCS, 00136 Rome, Italy; augusto.fusco@policlinicogemelli.it (A.F.); stefano.bonomi@guest.policlinicogemelli.it (S.B.); cristina.cuccagna@policlinicogemelli.it (C.C.); 2 Rehabilitation Medicine and Physiotherapy Unit, Humanitas Cellini Clinic, 10152 Turin, Italy; dario.gatto90@gmail.com; 3 Department of Neurosciences, Catholic University of the Sacred Heart, 00136 Rome, Italy; 4 Department of Geriatrics and Orthopaedics, Catholic University of the Sacred Heart, 00136 Rome, Italy; gianpaolo.ronconi@unicatt.it (G.R.); claudia.lombardo028@gmail.com (C.L.); 5 Physical and Rehabilitation Medicine, Department of Ageing, Orthopaedic and Rheumatological Sciences, Fondazione Policlinico Universitario “A. Gemelli” IRCCS, 00136 Rome, Italy

**Keywords:** focal muscle vibration, spasticity, severe acquired brain injury, disorder of consciousness, rehabilitation, Disability Rating Scale, Modified Ashworth Scale

## Abstract

**Background**: Spasticity significantly impairs functional recovery after severe acquired brain injury. Current management methods predominantly rely on pharmacological interventions, which can cause substantial side effects or require invasive medical procedures in refractory cases. Focal muscle vibration, a noninvasive technique that applies mechanical vibrations to muscle–tendon units and alters spinal and cortical excitability via proprioceptive pathways, has been effective in reducing spasticity in subjects with stroke. However, there is limited data to support focal muscle vibration as a viable option for improving functional recovery in patients with severe acquired brain injury. **Objectives**: To evaluate the clinical effects of adding focal muscle vibration to standard physiotherapy compared with standard physiotherapy alone in patients with severe acquired brain injury and spastic hypertonia. **Methods**: Twenty-four patients were randomly assigned to receive focal muscle vibration in addition to standard care (*n* = 12) or standard care alone (*n* = 12) for 3 weeks. Assessments were conducted at baseline, immediately after physiotherapy, and 3 weeks after physiotherapy. The outcomes assessed included the Modified Ashworth Scale, Disability Rating Scale, Modified Barthel Index, and three pain measures. **Results**: A significant reduction in spasticity was observed in the focal muscle vibration group, as indicated by the Modified Ashworth Scale scores (*p* = 0.014). Disability Rating Scale scores demonstrated a statistically significant decrease in disability ratings at the end of treatment (*p* = 0.002) and during the follow-up phase (*p* = 0.002). Between-group comparisons of change scores revealed a statistically significant improvement in disability ratings in the focal muscle vibration group during the treatment phase (*p* = 0.011). Significant functional gains were noted on the Disability Rating Scale, which persisted at the follow-up evaluation. **Conclusions**: Focal muscle vibration reduces muscle spasticity and improves functional status in patients with severe acquired brain injury during inpatient rehabilitation. Future studies with larger sample sizes, blinded assessments, and stratified randomization are needed to verify these findings and develop standardized treatment protocols for this underserved population.

## 1. Introduction

Spasticity is a velocity-dependent motor disorder resulting from an impaired tonic stretch reflex and is characterized by resistance to passive stretch [1]. This impairment leads to abnormal alterations in the rheological properties of the muscles, including increased stiffness, fibrosis, and muscle atrophy [2,3].

Current rehabilitative treatments address spasticity using a multimodal approach, including physiotherapy and, when necessary, focal or systemic intervention. The Royal College of Physicians’ national guidelines state that intramuscular botulinum toxin type A is an established and well-tolerated method for treating focal spasticity as part of a coordinated multidisciplinary rehabilitation program with clearly defined goals and plans for post-injection management [4]. Treatments based on botulinum toxin may have certain limitations that restrict their applicability. Patients with compromised respiratory status or pre-existing swallowing difficulties, which are common in severe brain injury, require close monitoring due to the risk of worsening dysphagia and respiratory function following injection [5]. Furthermore, 5–10% of patients may develop neutralizing antibodies over time, leading to secondary non-response [6], while primary non-responders (5–9%) may fail to respond due to established contractures or musculoskeletal complications [7]. In subjects with severe acquired brain injury (sABI) and/or disorders of consciousness, practical challenges include difficulty in obtaining cooperation for precise muscle targeting and the potential for respiratory compromise in already vulnerable individuals.

Although rehabilitation often relies on active and passive mobilization, device-delivered physical modalities can serve as useful adjuncts to standard care [8,9,10]. Focal muscle vibration (FMV) is a targeted mechanical stimulation that may complement conventional rehabilitation methods. FMV is a noninvasive intervention for spasticity that uses mechanical oscillations to apply vibrational forces directly to the muscles or tendons [11].

FMV delivers low-amplitude, high-frequency mechanical oscillations that primarily activate muscle spindles and Golgi tendon organs, transmitting proprioceptive signals to the central nervous system and modulating both spinal and cortical excitability [12,13]. This phenomenon is referred to as the “driving phenomenon,” and it facilitates the activation of specific afferent fibers and the stimulation of specific brain areas [14]. Additionally, the effects of FMV persist over time owing to long-term potentiation [15]. The therapeutic basis for the effect of FMV in treating spasticity is that repetitive vibrations stimulate muscle spindles and inhibit the stretch reflex [16].

The clinical potential of vibratory stimulation in rehabilitation has been explored for over four decades [17]. The mechanisms underlying spasticity reduction involve multiple levels: at the spinal level, repetitive proprioceptive input modulates presynaptic inhibition and persistent inward currents [18]; at the cortical level, FMV induces long-term increases in intracortical inhibition, promoting agonist–antagonist rebalance and improved joint impedance control [15,19].

Existing evidence on FMV has been generated almost exclusively in chronic stroke populations with preserved consciousness and residual voluntary motor activity [11]. Different studies have demonstrated that muscular vibration can assist physiotherapy in reducing spasticity and enhancing function during rehabilitation [20,21]. Preliminary evidence also suggests the feasibility of upper extremity vibration in individuals with tetraplegia due to spinal cord injury, demonstrating acceptability and spasticity reduction after a single session [22]. Patients with sABI are frequently unable to cooperate with clinical assessments, actively participate in rehabilitation, or generate voluntary muscle contractions considered optimal during vibratory stimulation. Furthermore, the sedative and cognitive-impairing side effects of systemic spasmolytic medications may diminish arousal and the level of consciousness, and no specific guidelines currently exist for spasticity management in this population [23]. Consistent with a most recent literature review on FMV [14], no randomized controlled trial has previously investigated FMV specifically in patients with sABI (a broader and more heterogeneous population than stroke, encompassing traumatic, vascular, and other etiologies) undergoing inpatient intensive rehabilitation. To the best of our knowledge, this study is the first to provide controlled evidence on FMV efficacy in this severely impaired population. The complexity of the clinical features of people with sABI determines a wide variability of the outcomes that cannot be extrapolated from stroke-based literature.

Therefore, our study aimed to evaluate the feasibility and clinical effects of adding intensive focal muscle vibration to standard physiotherapy versus physiotherapy alone on spasticity (primary aim), pain, and functional status (secondary aim) in patients with sABI. We explicitly designed this study as a pilot and exploratory trial to obtain preliminary estimates of the potential effects of FMV, given the complete absence of randomized controlled evidence on FMV in this severely impaired inpatient population.

## 2. Materials and Methods

### 2.1. Study Design

This was a prospective randomized pilot study with a control group (registration number: NCT05464160, clinicaltrials.gov). Due to the nature of the intervention, blinding of patients and treating therapists was not possible. Outcome assessors were also not blinded to the group allocation, while the statistical analyses were performed by a researcher not involved in patient recruitment or treatment delivery. We designed this study as a feasible pilot study because of the scarcity of literature on this population of patients with sABI in relation to the type of intervention and the delivery of treatment in an inpatient setting. Accordingly, we planned a sample size of 24 patients, divided into two equal groups of 12 patients. Simple randomization was used to allocate patients to the two groups in a 1:1 ratio. The present study was conducted from October 2021 to October 2024 and included individuals with sABI who were admitted to our academic hospital and who met the inclusion criteria.

Patients were included in this pilot study according to the following inclusion criteria: (i) sABI due to vascular or traumatic etiology; (ii) latency from the acute event of at least 20 days; and (iii) spasticity of the upper and/or lower limbs, ranging from 1+ to 3 on the Modified Ashworth Scale (MAS) [24,25].

In contrast, patients with the following characteristics were excluded: (i) deep vein thrombosis; (ii) venous access on the limbs to be treated; (iii) oncological diseases; (iv) epilepsy; (v) open skin lesions and/or local infections and/or sepsis; (vi) recent treatment with botulinum toxin (within three months); and (vii) ongoing treatment with systemic muscle relaxant drugs (baclofen, tizanidine, and benzodiazepines). Patients included in the study were randomized according to a computer-generated 1:1 allocation ratio using a random procedure with PASS2021 (version 21.0.3) software.

### 2.2. Interventions

Patients were randomly assigned to the experimental (G-FMV) and control (G-CON) groups. The G-FMV group received focal vibratory intervention in addition to conventional treatment for spasticity (passive rehabilitation in bed, manual therapy, muscle stretching, neuromotor facilitation activity, and orthosis use) 7 days a week for a total of 3 weeks. Each vibration intervention comprised four sessions lasting five minutes each, with one minute of rest (a total of 21 min) and three minutes of rest, and a frequency of 100 Hz. Vibratory stimulation was applied to the agonist muscles of the lower and/or upper limbs for single or multiple areas according to the clinical examination supporting the intervention. FMV was applied using a noninvasive device, EVM-EVO (Endomedica Srl, Rome, Italy), which exploits the influence of acoustic waves on biological tissues by quickly modulating air pressure and depression within the range of 30–300 Hz to stimulate the neuromuscular components. Transducers were applied to the myotendinous junction of the target muscle. Specifically, treatment was primarily delivered to the biceps brachii, quadriceps, and triceps surae muscles.

For 3 weeks, the patients in the G-CON group received only standard rehabilitation treatments for the same duration as the sessions in the G-FMV group.

The theoretical framework was formulated based on the frequency, intensity, time, and type of intervention (FITT) principles of physical rehabilitation therapeutic modalities to create a systematic and suitable treatment protocol for the study. The variables can be adjusted to meet the functional, physiological, and psychological demands in accordance with the specific aims of the study [26].

**Figure 1:**
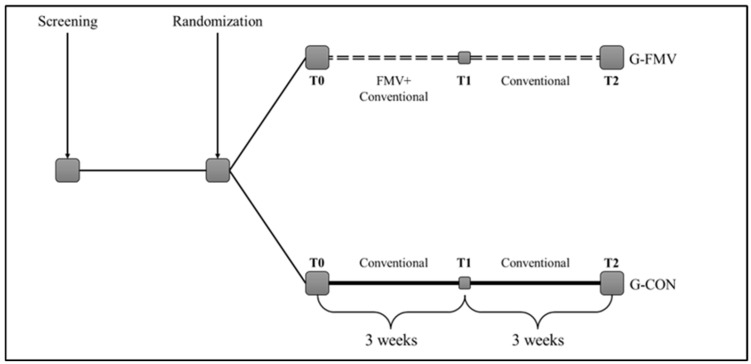
Design of the study.

Figure 1 illustrates the study design.

### 2.3. Assessments

Clinical assessments were performed at onset (T0, baseline), 3 weeks after treatment (T1), and at follow-up. At baseline, the following information was collected: age, sex, schooling, comorbidities, latency from the acute event, and diagnosis. At T0, T1, and T2, the following rating scales were used: (i) Modified Ashworth Scale (MAS), (ii) Disability Rating Scale (DRS), (iii) Modified Barthel Index (mBI), (iv) Critical Care Pain Observation Tool (C-POT), (v) Nociception Coma Scale—Revised (NCS-R), and (vi) Pain Assessment in Advanced Dementia (PAINAD).

The MAS is the most commonly used clinical scale for measuring muscle tone. The scores were 0, 1, 1+, 2, 3, and 4 [27].

The DRS assesses a patient’s disability through four domains: (i) vigilance, awareness, and responsiveness; (ii) cognitive ability for self-care activities; (iii) functional level; and (iv) employability. The score ranges from 0 to 30, with 0 (no disability) and 30 (death). Ten categories can be identified based on the DRS score, from “no disability” (category 1) to “death” (category 10) [28].

The mBI is used to assess a patient’s functional independence. It assesses 10 items, and the overall score ranges from 0 (total dependence) to 100 (total independence) [29].

Pain assessment in patients with sABI is a significant clinical challenge because of the heterogeneity of consciousness levels in this population. Patients may range from unresponsive wakefulness syndrome to an emerging state after a disorder of consciousness, and many of them are unable to communicate their pain verbally. Given that no single pain assessment tool has been validated for all levels of consciousness impairment, we adopted a multimodal approach that used three complementary behavioral observation scales. This strategy allowed for a comprehensive pain evaluation across the spectrum of awareness levels present in our study population, maximizing the likelihood of detecting nociceptive responses, regardless of the patient’s cognitive status.

The Critical Care Pain Observation Tool (C-POT) is a behavioral observation scale specifically developed for the assessment of pain in critically ill patients unable to self-report. This tool evaluates four distinct domains: facial expression, body movement, muscle tension, and compliance with the ventilator (for intubated patients) or vocalization (for non-intubated patients). Each domain is assigned a score ranging from 0 to 2, resulting in a total score between 0 and 8, with higher scores indicating increased pain intensity [30].

The NCS-R is a behavioral observation tool specifically developed to assess nociception in patients with disorders of consciousness. It evaluates three domains: motor response, verbal response, and facial expressions. Each item is scored from 0 to 3, with a total score ranging from 0 to 9. Higher scores suggest a greater behavioral response to noxious stimulation. The NCS-R has been correlated with cerebral metabolism in pain-processing regions, supporting its validity as a measure of nociceptive processing in noncommunicative brain-injured patients [31,32].

The PAINAD scale is a five-item observational tool originally developed for pain assessment in patients with advanced dementia who are unable to communicate verbally. It evaluates breathing patterns, negative vocalizations, facial expressions, body language, and consolability. Each item is scored from 0 to 2, with a total score ranging from 0 to 10. Higher scores indicate more observable pain behaviors [33].

### 2.4. Statistical Analysis

The clinical and demographic features of the participants are reported using descriptive statistics. Continuous data are presented as mean ± standard deviation, and categorical data as frequencies and percentages.

The MAS was transformed from an ordinal to a numerical scale as follows: 0 = 0; 1 = 1; 1+ = 2; 2 = 3; 3 = 4; and 4 = 5. This conversion allowed both parametric and nonparametric statistical assessments without losing the scale’s ordinal properties.

Normality of the data was assessed using the Shapiro–Wilk test, which demonstrated that most variables did not follow a normal distribution (*p* < 0.05). Consequently, nonparametric statistical tests were used for all comparisons among groups: the Mann–Whitney U test for comparisons between two independent groups, the Friedman test for general longitudinal comparisons, and the Wilcoxon signed-rank test for comparisons between any two time points. A Bonferroni-corrected significance level of *p* < 0.025 was used for post hoc analyses to account for multiple comparisons.

## 3. Results

### 3.1. Sample Characteristics

**Figure 2:**
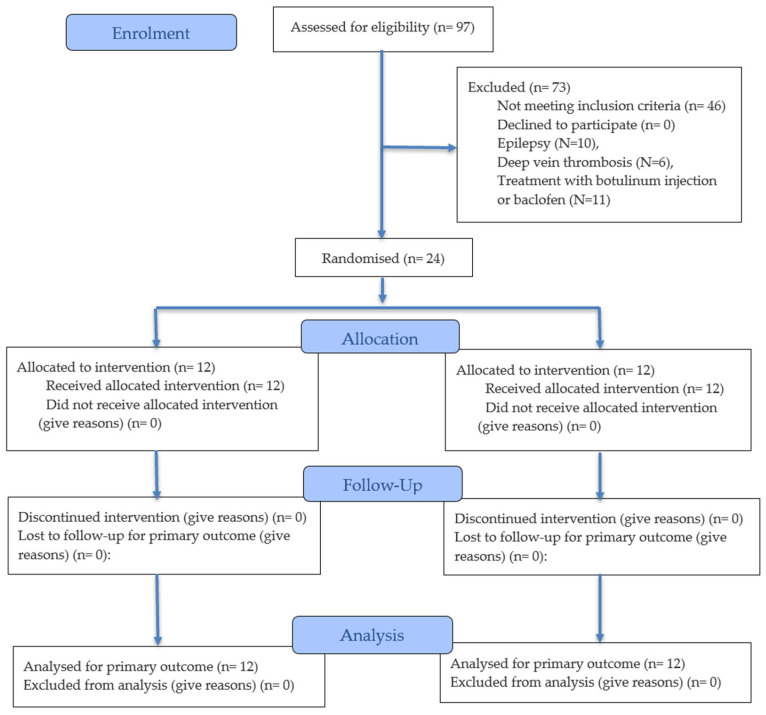
CONSORT 2025 flow diagram.

Twenty-four patients with sABI were enrolled in the study and randomly allocated to the G-FMV (*n* = 12) or the G-CON (*n* = 12). A CONSORT-style flow diagram is provided (Figure 2) for the sake of clarity. None of the patients were lost to follow-up, and complete data were available for all 24 participants at all three assessment time points (T0, T1, and T2).

The demographic and clinical characteristics of the participants are presented in Table 1.

The mean age of the participants was 57.2 ± 9.2 years in the G-FMV and 56.7 ± 21.6 years in the G-CON. The proportion of males was 58.3% in G-FMV and 66.7% in G-CON.

Regarding etiology, ischemic stroke was more frequent in the G-FMV group (50.0% vs. 8.3%), whereas traumatic brain injury was more common in the G-CON group (33.3% vs. 8.3%). The mean latency from the acute event to enrollment was 210.3 days in G-FMV and 307.1 days in G-CON.

Regarding the level of consciousness, the majority of patients in the G-FMV group emerged from a disorder of consciousness (75.0%), while the G-CON group showed a more heterogeneous distribution, as shown in Table 1.

Upper limb muscles were treated in most patients (83.3% and 66.7% in the G-FMV and G-CON, respectively).

Although no statistically significant differences were found between groups for demographic and clinical variables at baseline (all *p* > 0.05), clinically relevant imbalances in etiology distribution and latency from the acute event were observed. This should be considered when interpreting the results.

### 3.2. Baseline Comparison of Outcome Measures

A comparison of the results from the T0 data is shown in Table 2. Both groups were similar as measured by the MAS (G-FMV: 2.50 ± 0.67 vs. G-CON: 2.50 ± 0.80, *p* = 0.867), DRS (G-FMV: 17.42 ± 5.04 vs. G-CON: 20.33 ± 2.71, *p* = 0.092), NCS-R (*p* = 0.952), C-Pot (*p* = 0.077), and PAINAD (*p* = 0.163).

A significant difference was observed at baseline in mBI (G-FMV: 11.25 ± 17.52 vs. G-CON: 0.00 ± 0.00, *p* = 0.003). Therefore, baseline imbalances in mBI should be considered when comparing group differences.

### 3.3. Longitudinal Within-Group Analysis

The Friedman test revealed a significant temporal trend in G-FMV for the three measured outcomes: MAS (χ^2^ = 8.72; *p* = 0.013), DRS (χ^2^ = 15.45; *p* < 0.001), and mBI (χ^2^ = 10.56; *p* = 0.005). However, the G-CON data revealed only a significant temporal trend in mBI (χ^2^ = 7.71; *p* = 0.021), with no significant trends observed for MAS (*p* = 0.174) or DRS (*p* = 0.184). In addition, the Friedman test did not reveal any significant longitudinal trends in the pain-related outcomes (NCS-R, C-Pot, and PAINAD) in either treatment group (Table 3).

### 3.4. Post Hoc Pairwise Comparisons

Wilcoxon signed-rank post hoc analyses were conducted on outcomes that were statistically significant in Friedman tests. The G-FMV group demonstrated a statistically significant improvement in the MAS at the end of the intervention compared to the beginning (T0–T2: *p* = 0.014) and a statistically significant improvement in the Disability Rating Scale (DRS) scores at two time points (T0–T1: *p* = 0.002; T0–T2: *p* = 0.002). For mBI, although the Friedman test indicated a significant temporal trend in the G-FMV group, post hoc pairwise comparisons did not reach statistical significance after Bonferroni correction (T0–T1: *p* = 0.031; T0–T2: *p* = 0.035; threshold *p* < 0.025). No statistically significant differences were observed in the G-Con group when comparing the pre-treatment period with either of the two post-treatment periods (Bonferroni-corrected *p* < 0.025) (Table 4).

### 3.5. Between-Group Comparison of Change Scores

**Figure 3:**
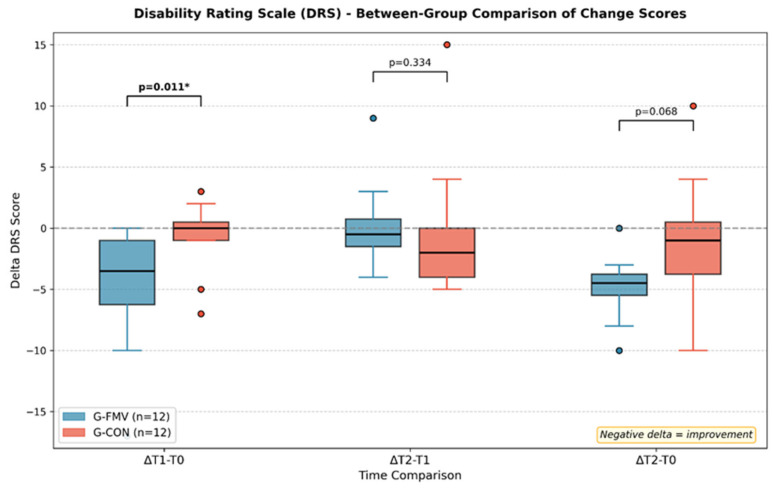
Between-group comparison of DRS change scores. Box plots show the distribution of delta values for each time comparison. G-FMV (blue) showed a significantly greater improvement during the treatment phase (ΔT1−T0, *p* = 0.011). Dotted lines represent to highlight point 0 in terms of change. *: significant values.

**Figure 4:**
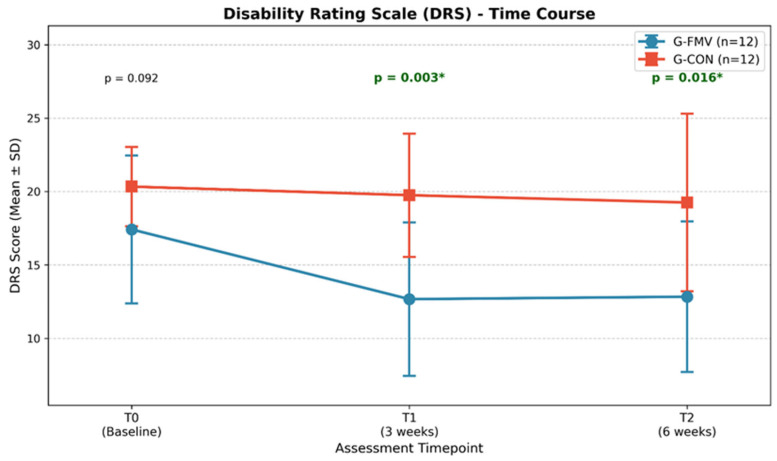
Time course of the Disability Rating Scale (DRS) scores. Values are expressed as mean ± SD. G-FMV showed a marked decrease in DRS scores during the treatment phase (T0 to T1), which was maintained at follow-up. *: significant values.

A between-group comparison of change scores (delta) revealed a significant difference in DRS scores during the treatment phase (ΔT1−T0). G-FMV showed a mean reduction of −4.75 ± 4.90 points, compared to −0.58 ± 2.84 points in G-CON (*p* = 0.011). The overall change from baseline to follow-up (ΔT2−T0) also showed a trend favoring G-FMV (−4.58 ± 2.91 vs. −1.08 ± 5.16, *p* = 0.068). The between-group comparison of delta scores is shown in Figure 3, and the time course of the DRS scores is illustrated in Figure 4.

**Figure 5:**
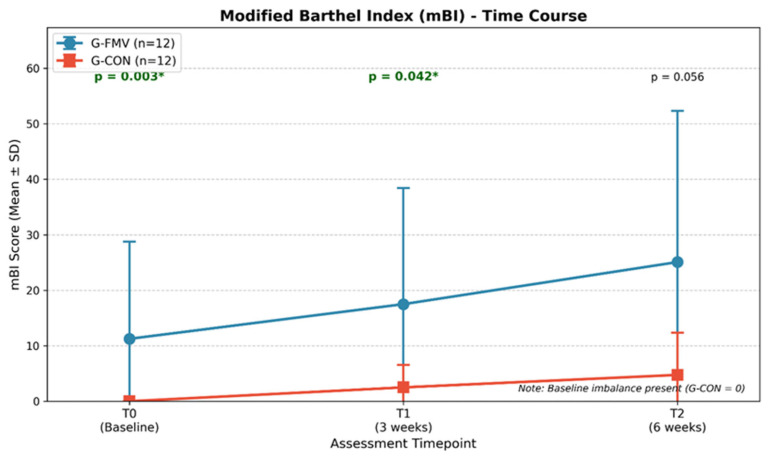
Time course of Modified Barthel Index (mBI) scores. Values are expressed as mean ± SD. Both groups showed improvement over time, but the baseline imbalance (G-CON starting at 0) limited the between-group comparisons. *: significant values.

No significant between-group differences were observed in the change scores for mBI, NCS-R, C-Pot, or PAINAD. However, a trend toward greater improvement in G-FMV was observed for mBI (ΔT2−T0:13.83 ± 23.30 vs. 4.75 ± 7.61, *p* = 0.280), although this result should be interpreted cautiously because of the baseline imbalance. The time course of the mBI scores is shown in Figure 5.

## 4. Discussion

The results of this study indicate that focal muscle vibration (FMV) applied to the agonist muscles of spasticity is a clinically feasible method for treating severely impaired individuals in the chronic phase of acquired brain injury. Notably, there was a statistically significant reduction in spasticity and a corresponding improvement in functional ability in patients treated with FMV. In contrast, no similar measurable improvements were observed in the control group. Functional improvements in patients treated with FMV were observed after treatment and remained stable during the follow-up period, indicating that the beneficial effects of FMV persisted after the termination of the treatment.

The level of independent functioning in the daily lives of all participants increased over time; however, the large difference between the two groups at the onset limits the significance of this finding. No statistically significant changes were observed when any of the three pain assessment methods were used. The lack of response likely represents a limitation of the available pain assessment tools for use in the complex patient population of individuals with severe acquired brain injury and varying states of awareness and/or consciousness. Improved pain assessment tools are needed for use in severely injured individuals across the spectrum of brain injury severity and state of awareness or consciousness.

### 4.1. Effects on Spasticity

The significant reduction in spasticity observed in the G-FMV group was consistent with the results of previous studies investigating the effects of FMV in neurological populations [22,35]. Our findings extend this evidence to patients with sABI.

In clinical terms, the mean MAS reduction in the G-FMV group from 2.50 to 1.50 corresponds approximately to a shift from a marked to a slight increase in muscle tone. The degree of change in muscle tone observed in individuals with severe acquired brain injury has significant implications for their medical treatment; it facilitates joint passive movement (a method used by therapists), lowers the incidence of pressure sores and contractures, reduces the number of nurses needed to position patients and assist them with personal hygiene activities, and decreases the amount of medication required for systemic side effects. These increases in ease of care and comfort provide additional positive aspects in terms of quality of care for patients who cannot independently participate in therapy, even when no measurable improvements in functional activity have occurred.

Beyond immediate reflex modulation, FMV has been hypothesized to induce neuroplastic changes resembling long-term potentiation [36]. In the present study, clinical improvements persisted at the three-week follow-up. The repetitive and structured sensory input provided by FMV may help maintain sensorimotor circuitry and facilitate functional recovery [14]. However, no neurophysiological or neuroimaging assessments were conducted. Future studies incorporating such measures are needed to determine whether neuroplastic mechanisms contribute to the effects of FMV in patients with sABI.

A critical consideration in interpreting our results is the latency period from acute events to study enrollment. In our sample, G-FMV had a mean latency of approximately 7 months, whereas G-CON had a longer latency of approximately 10 months, although this difference was not statistically significant. This timing places our patients in the subacute-to-chronic phase of recovery, when spasticity has typically become established and stabilized. According to the natural history of spasticity after brain injury, muscle tone abnormalities typically emerge 1–6 weeks after the acute event and plateau at approximately 3–6 months [37,38].

The observation that FMV led to significant reductions in spasticity in patients, on average, 7–10 months post-injury, suggests that this intervention can be effective even when spasticity has become chronic. This is clinically important because chronic spasticity is often more resistant to treatment and may be associated with secondary musculoskeletal complications such as contractures, pain, and reduced range of motion [39]. Previous studies have suggested that FMV may be particularly effective in chronic conditions because it targets both neural and non-neural components of spasticity, including the viscoelastic properties of the muscle tissue [13]. Mechanical stimulation provided by vibration may help maintain muscle fiber length and compliance, thereby counteracting the fibrotic changes that develop during prolonged muscle immobilization.

However, the wide variability in latency within our sample (ranging from approximately 1 to 18 months) may have introduced heterogeneity in the treatment response. Some evidence suggests that neuroplasticity-based interventions may be more effective when initiated early during the critical window of enhanced brain plasticity [40]. Subgroup analyses based on chronicity were not feasible in our small sample but would be valuable in future larger studies to determine whether FMV efficacy varies with time since the injury.

### 4.2. Effects on Functional Status and Pain

The Disability Rating Scale demonstrated sensitivity to improvement within our sample, as evidenced by the identification of real functional gains, which were documented through improvements in DRS scores. Although there was no statistically significant difference in the baseline DRS scores, the data showed a trend toward greater initial disability levels in the G-CON, which may have influenced the observed improvements between groups. The mean DRS reduction of approximately 4.75 points in the G-FMV group during the treatment phase corresponds to a shift of nearly one full disability category. These changes are directly applicable to clinical practice and should be considered when evaluating the intensity of care that should be provided, whether patients would benefit from continued inpatient rehabilitation, or what type of support caregivers will need. Additionally, with the continuation of significant improvements at the follow-up assessment, we may argue that the changes were not simply temporary effects of the treatment sessions but rather longer-term, more sustainable changes in each participant’s disability ratings.

The Modified Barthel Index (mBI) showed a statistically significant temporal trend in the Friedman test for both groups. However, post hoc pairwise comparisons in the G-FMV group did not reach statistical significance after the Bonferroni correction. Furthermore, a statistically significant baseline disparity was observed, with all subjects in the G-CON group scoring “zero” at study entry, indicating a pronounced floor effect. The mBI has been identified as having limitations in terms of its discriminative capability (i.e., ability to differentiate levels of impairment) and responsiveness, particularly with regard to measuring function at the most impaired level of the spectrum [41]. This psychometric feature of the mBI limits the measurement of the gains in functional status when patients are clustered at the lowest measurable value, suppressing the reported effects of interventions regardless of their real impact.

Secondly, in our small pilot sample, this baseline floor effect was most likely due to random variation for this outcome despite adequate randomization procedures being performed. This may reflect the inherent risk of chance imbalances in trials with small sample sizes. As a result, the observed between-group differences in mBI compromise the interpretability of between-group comparisons as treatment effects, and their clinical meaning should be considered with caution. Future studies should employ stratified or covariate-adaptive randomization based on baseline functional status, increase the sample size, and combine the mBI with additional functional outcome measures.

To assess pain, we utilized three separate assessments: the NCS-R, which is designed for use with disorders of consciousness; the PAINAD, which has been used to assess pain in nonverbal patients; and the C-Pot, which assesses the capacity for communication. The use of multiple assessments was required to accommodate the vast degree of variance in the level of consciousness among the participants in our study. Although there were baseline differences in pain assessment, none were statistically significant. The lack of measurable change over time using each of the assessment tools was most likely due to the limitations associated with each tool’s ability to measure the pain experience in patients with sABI. Improved assessment tools to measure the pain experience in these patients remain an unmet clinical need [31,42].

### 4.3. Clinical Implications

Spasticity does not represent the full extent of complications in individuals with sABI. During a period of immobility, they are likely to lose significant amounts of muscle mass due to prolonged periods of inactivity, their bones begin to deplete in density (which increases the risk of fractures), and their soft tissues are shortened. In addition to these physical changes, brain injury may cause individuals to exhibit abnormal behaviors (such as posturing), have difficulty maintaining appropriate autonomic function (i.e., “dysautonomia”), and have an increased risk of developing various infections, pressure ulcers, and thromboembolic events [43]. The progression from spasticity to contracture and ultimately to further immobility and decreased functionality is greatly accelerated by spasticity [44].

FMV has several practical advantages over other spasticity treatment options. First, FMV is noninvasive and has no systemic side effects [45]. Second, FMV can be used in patients who are unable to cooperate with treatment; therefore, FMV can be used regardless of the level of consciousness of the patient. Pazzaglia et al. demonstrated that focal muscle vibration induced significant modifications in heart rate variability in patients with sABI, suggesting that proprioceptive stimulation can modulate autonomic nervous system activity [46]. FMV may exert systemic effects through central mechanisms and contribute to overall clinical improvement by addressing dysautonomia. Future studies should be conducted to evaluate these outcomes.

### 4.4. Limitations and Perspectives

The results of this study should be interpreted in light of its limitations. The most important limitation of this research project is the number of participants involved, which was understood and anticipated as part of pilot studies. Thus, our investigation was specifically designed as an exploratory clinical trial to obtain initial estimates of effect sizes, assess the feasibility of this type of intervention in an inpatient neurological rehabilitation program, and determine methodological issues; therefore, it does not provide a definitive measure of effectiveness. The effects measured in our study, especially the between-group DRS difference and the within-subject MAS decrease, will serve as the foundation for future confirmatory clinical trials. As such, the results of our investigation should be viewed as hypothesis-generating.

Moreover, the small sample size limited the statistical power and contributed to the baseline imbalances observed for some outcomes between groups, such as the mBI at baseline. The small number of enrolled patients made stratified randomization impractical. The heterogeneity of our population, while reflecting the clinical practice in a neurorehabilitation ward, may lead to differential treatment responses in specific subgroups of patients and represent a potential confounding factor. It is also possible that the study design contributed to the baseline variability. One potential reason is the large difference in time between the acute event and study enrollment, as indicated by the high standard deviations. This variability may have increased sample heterogeneity and influenced the treatment response. Future larger studies should consider stratified randomization based on important prognostic factors, such as etiology, level of consciousness, and baseline functional status in order to reduce the risk of bias. In addition, rigorously designed controlled observational studies, including prospective cohort and case–control studies, could be useful alongside randomized trials in this population. These studies may improve external validity, allow exploration of predictors of treatment response, and guide the choice of target subgroups and outcome measures for future randomized controlled trials [47,48].

The relatively short follow-up duration (three weeks) may be inadequate to assess long-term effectiveness or stability. Simultaneously, the shorter timeframe may have contributed to the lack of missing data. Both measurement instruments (mBI and pain assessment tools) used in the study are vulnerable to floor effects in participants who experience extreme disabilities. These limitations cannot be overstated when evaluating our reported outcomes, particularly for mBI. It should be noted that the issue of floor effects in functional scales is a well-recognized phenomenon in patients with acquired brain injury undergoing rehabilitation. Scheuringe and colleagues reported that the proportion of patients scoring at the minimum on functional independence measure subscales ranges from 22% to nearly 48%, leading to the conclusion that most of the functional tools of assessment may lack sufficient discriminative capacity in the most severely impaired patients [49]. Therefore, future studies should consider differences in sensitivity to change at each end of the scale when choosing measurement instruments and using randomization procedures that include stratification. Future larger studies should be designed with a priori sample size determinations, longer follow-up extending beyond hospital discharge, instruments with validated responsiveness in disorders of consciousness populations, and a form of assessor blinding to minimize detection bias.

Despite these limitations, we believe that this study provides valuable preliminary evidence of FMV efficacy in sABI. Current spasticity management in these individuals relies heavily on pharmacological interventions with a high risk of side effects, and invasive procedures are reserved for refractory cases. A noninvasive, well-tolerated intervention capable of improving spasticity and global function can address this clinical need.

## 5. Conclusions

In patients with severe acquired brain injury in the chronic phase of recovery, focal muscle vibration has been shown to be a useful treatment method for decreasing spasticity and enhancing overall function, as measured by a global assessment. The improvements were clinically significant and persisted at follow-up, consistent with previously described mechanisms of neural plasticity. However, the lack of influence of FMV on pain measures demonstrates the need to develop better assessment tools for this population. FMV has demonstrated potential as a noninvasive adjunct to the rehabilitation process for individuals who have experienced severe brain injury, and it is recommended that future research investigate FMV using larger controlled studies with more patients.

## Figures and Tables

**Table 1:** Descriptive statistics of the sample (*N* = 24).

Variable	G-FMV (*n* = 12)	G-CON (*n* = 12)	*p*-Value
**Age (years), mean ± SD**	57.2 ± 9.2	56.7 ± 21.6	0.371
**Sex, *n* (%)**			1.000
Male	7 (58.3%)	8 (66.7%)	
Female	5 (41.7%)	4 (33.3%)	
**Etiology, *n* (%)**			0.095
Hemorrhagic stroke	4 (33.3%)	4 (33.3%)	
Ischemic stroke	6 (50.0%)	1 (8.3%)	
TBI	1 (8.3%)	4 (33.3%)	
Other (cardiogenic hypoxia and encephalopathies)	1 (8.3%)	3 (25.0%)	
Latency (days), mean ± SD	210.3 ± 184.9	307.1 ± 219.1	0.214
**DOC Level, *n* (%)**			0.231
0—Coma	1 (8.3%)	0 (0.0%)	
1—UWS	0 (0.0%)	1 (8.3%)	
2—MCS−	0 (0.0%)	3 (25.0%)	
3—MCS+	2 (16.7%)	2 (16.7%)	
4—Emerging DOC	9 (75.0%)	6 (50.0%)	
**Treated muscle, *n* (%)**			0.491
Upper limb	10 (83.3%)	8 (66.7%)	
Lower limb	2 (16.7%)	3 (25.0%)	
Other	0 (0.0%)	1 (8.3%)	

SD: standard deviation; TBI = traumatic brain injury; DOC = disorder of consciousness; UWS = unresponsive wakefulness syndrome; MCS = minimally conscious state. The difference +/− was according to the Coma Recovery Scale—Revised (CRS-r) [34]. Mann–Whitney U test for continuous variables, Fisher’s exact test, and ‡ chi-square test.

**Table 2:** Comparison of outcomes at baseline (T0).

Outcome	G-FMV (*n* = 12)	G-CON (*n* = 12)	*p*-Value
**MAS**	2.50 ± 0.67	2.50 ± 0.80	0.867
**DRS**	17.42 ± 5.04	20.33 ± 2.71	0.092
**mBI**	11.25 ± 17.52	0.00 ± 0.00	0.003 *
**NCS-R**	2.83 ± 3.07	2.83 ± 2.62	0.952
**C-Pot**	2.67 ± 2.61	1.00 ± 1.91	0.077
**PAINAD**	2.50 ± 3.06	0.83 ± 1.59	0.163

Values are expressed as the mean ± SD. Mann–Whitney U test. * *p* < 0.05. MAS, Modified Ashworth Scale (converted: 0 = 0, 1 = 1, 1+ = 2, 2 = 3, 3 = 4, 4 = 5); DRS, Disability Rating Scale; mBI, Modified Barthel Index; NCS-R, Nociception Coma Scale—Revised; C-Pot, Critical Care Pain Observation Tool; PAINAD, Pain Assessment in Advanced Dementia.

**Table 3:** Longitudinal analysis—within-group (Friedman test).

Outcome	Group	T0	T1	T2	χ^2^	*p*-Value
**MAS**	G-FMV	2.50 ± 0.65	2.08 ± 0.49	1.50 ± 0.76	8.72	0.013 *
	G-CON	2.50 ± 0.76	2.42 ± 0.64	2.08 ± 1.04	3.50	0.174
**DRS**	G-FMV	17.42 ± 4.82	12.67 ± 5.01	12.83 ± 4.91	15.45	<0.001 *
	G-CON	20.33 ± 2.59	19.75 ± 4.02	19.25 ± 5.79	3.39	0.184
**mBI**	G-FMV	11.25 ± 16.77	17.50 ± 20.04	25.08 ± 26.10	10.56	0.005 *
	G-CON	0.00 ± 0.00	2.50 ± 3.91	4.75 ± 7.28	7.71	0.021 *
**NCS-R**	G-FMV	2.83 ± 2.94	2.50 ± 2.36	2.58 ± 1.89	0.28	0.871
	G-CON	2.83 ± 2.51	3.08 ± 2.47	3.83 ± 2.03	1.13	0.568
**C-Pot**	G-FMV	2.67 ± 2.49	2.42 ± 2.14	2.75 ± 1.69	0.00	1.000
	G-CON	1.00 ± 1.83	1.75 ± 2.20	3.33 ± 2.53	3.31	0.191
**PAINAD**	G-FMV	2.50 ± 2.93	2.00 ± 1.96	2.42 ± 1.75	0.90	0.639
	G-CON	0.83 ± 1.52	1.42 ± 2.47	3.42 ± 3.04	2.82	0.244

Values are expressed as mean ± SD. * *p* < 0.05. Friedman test for repeated measures.

**Table 4:** Post hoc pairwise comparisons (Wilcoxon signed-rank test).

Outcome	Group	T0 vs. T1	T1 vs. T2	T0 vs. T2
**MAS**	G-FMV	0.188	0.113	0.014 *
	G-CON	1.000	0.312	0.250
**DRS**	G-FMV	0.002 *	0.766	0.002 *
	G-CON	0.797	0.344	0.414
**mBI**	G-FMV	0.031	0.102	0.035
	G-CON	0.125	0.281	0.062
**NCS-R**	G-FMV	0.766	0.672	0.812
	G-CON	0.875	0.500	0.328
**C-Pot**	G-FMV	0.875	0.684	0.922
	G-CON	0.250	0.219	0.047
**PAINAD**	G-FMV	0.688	0.422	0.914
	G-CON	0.750	0.156	0.062

Values are p-values from the Wilcoxon signed-rank test. * *p* < 0.025 (Bonferroni-corrected threshold).

## Data Availability

Data supporting the results are available upon request from the corresponding author.

## Abbreviations

The following abbreviations are used in this manuscript:

DOC	Disorders of Consciousness
FMV	Focal Muscle Vibration
sABI	Severe Acquired Brain Injury
MAS	Modified Ashworth Scale
G-FMV	Focal Muscle Vibration Group
G-CON	Control Group
FITT	Frequency, Intensity, Time, Type of intervention
DRS	Disability Rating Scale
mBI	Modified Barthel Index
C-POT	Critical Care Pain Observation Tool
NCS-R	Nociception Coma Scale—Revised
PAINAD	Pain Assessment in Advanced Dementia
TBI	Traumatic Brain Injury
UWS	Unresponsive Wakefulness Syndrome
MCS	Minimally Conscious State
